# After action review of the COVID-19 pandemic response in North West province, South Africa

**DOI:** 10.4102/sajid.v38i1.571

**Published:** 2023-12-18

**Authors:** John M. Tumbo, Indiran Govender, Doudou K. Nzaumvila

**Affiliations:** 1 Department of Family Medicine and Primary Health Care, Faculty of Medicine, Sefako Makgatho Health Sciences University, Pretoria, South Africa

**Keywords:** coronavirus disease 2019, COVID-19, after action review, pandemic, COVID-19 vaccination

## Abstract

**Background:**

Severe acute respiratory syndrome coronavirus 2 (SARS-CoV-2) caused coronavirus disease 2019 (COVID-19) pandemic with major disruptions globally. Northwest Province Department of Health (NWDoH) in South Africa set up comprehensive epidemiological emergency response plans for preventing, finding, containing and stopping the spread of COVID-19 in accordance with the *National Disaster Management Act*.

**Objectives:**

This After-Action Report (AAR) describes the provincial response to the pandemic from September 2020 to October 2022.

**Method:**

The AAR was conducted using the World Health Organization AAR methodology. Focus groups discussed five items: coordination, leadership and governance; epidemiology, surveillance and laboratory; case management and continuity of essential services; risk communication and community engagement and COVID-19 vaccination.

**Results:**

The timely establishment and activation of provincial intergovernmental and intersectoral coordinating structures led to effective coordination, resource mobilisation, leadership, decision-making and intervention. The effective communication in the department and other stakeholders resulted in improved surveillance data quality, timelier response and increased ownership of data. Dissemination, training and implementation of case management protocols ensured standardised case management. The multi-channel information dissemination targeting different audiences empowered people with real-time knowledge on the infection and encouraged health-seeking behaviours.

**Conclusion:**

The AAR demonstrated the importance of coordinated epidemiological, laboratory and communication response that requires significant public health reserve capacity in peacetime for rapid expansion in an emergency.

**Contribution:**

This review contributes to the body of knowledge emerging from the COVID-19 pandemic and provides guidance on enhanced public health response to future emergencies.

## Background

The severe acute respiratory syndrome coronavirus 2 (SARS-CoV-2) that causes coronavirus disease 2019 (COVID-19) was first detected in Wuhan, China in December 2019. Coronavirus disease 2019 has caused major disruptions and devastations across the world never witnessed since the 1918 H1N1 influenza pandemic. The COVID-19 outbreak in South Africa started on 05 March 2020 when the first case was confirmed, and the outbreak was subsequently declared a national disaster on 14 March. This imposed public health and social measures, including banning mass public gatherings, closure of all institutions of learning and limiting population movement. These early actions allowed critical capacities such as testing and health facility readiness to be scaled up.

As the first COVID-19 case was reported in South Africa, ongoing critical public health response capacities were established in all nine provinces. The Northwest Provincial COVID-19 Strategic Management Committee was formed to identify and implement advanced planning on community mitigation strategies and preparedness measures for the eventual impact on the healthcare sector. In accordance with the *Disaster Management Act* protocols and guidelines, the North West Department of Health (NWDoH) set up comprehensive, step-by-step emergency response plans for preventing, finding, containing and stopping the spread of COVID-19. As the Northwest Province (NWP) began to report an increase in COVID-19 cases in March 2020, healthcare systems intensified their epidemiology and response activities to address the rise in patients and fatalities. To share information and keep track of what was going on, the COVID-19 Strategic Management Committee set up weekly situational meetings and a daily scenario report.

**FIGURE 1:**
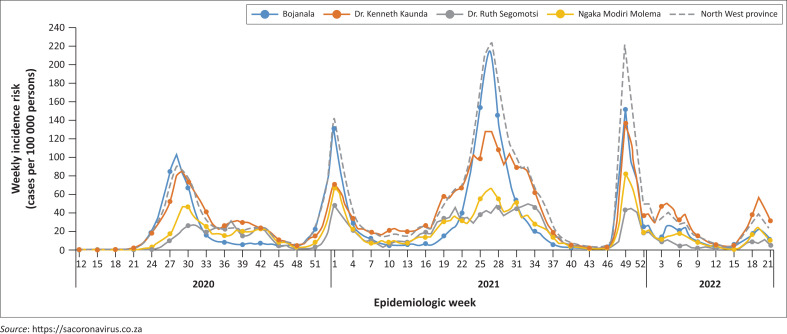
North west epidemiological curve.

Northwest Province with a population of 4.1 million had reported a total of 203 451 confirmed COVID-19 cases, 143 active cases and 5060 deaths as of 25 November 2022 (Figure 1).

Coronavirus disease 2019 vaccination rates remained low at 47.62% by November 2022 relative to the goal of vaccinating 70% of adults aged 18 and older. By the end of 2022 NWP, like the rest of the nation, was experiencing a period of low transmission. Even though testing, cases and deaths had significantly gone down in NWP, it is important to note that the COVID-19 pandemic was still going on.

The province conducted an intra-action review (IAR) in September 2020, which informed response strategies in the later part of the pandemic. This After-Action Report (AAR) addresses the response by the NWP to the novel COVID-19 pandemic from 18 September 2020, through 31 October 2022. This window aligns with the last IAR and the reduction of COVID-19 cases and hospitalisations across the NWP, and at the point where most health services were utilising day-to-day systems rather than emergency operations. The AAR offered the stakeholders in the province a forum to reflect on the response and identify best practices, gaps and lessons learned. It was also important to suggest ways to improve the NWDoH’s overall emergency response capabilities.

### Aim and objectives of the After-Action Report

The aim of this review was to describe the COVID-19 response undertaken in NWP, South Africa in order to better prepare for future pandemics and other disasters.

The AAR was a structured, qualitative review of interventions undertaken to respond to COVID-19 in the province since 2020. The specific objectives of the NWP AAR were threefold, namely:

1. To provide an opportunity to share experiences and collectively analyse the NWDoH’s response to COVID-19 by identifying best practices and challenges.
2. To document and apply lessons learnt from the response efforts to strengthen health systems and capacities for emergency preparedness and response.
3. To develop a plan for sustained vigilance, strengthening emergency preparedness and response and building health system resilience as part of recovery, resetting and recalibration.

## Methods

The AAR was conducted from 01 to 02 November 2022 in Rustenburg, NWP. The review followed the World Health Organization (WHO) AAR methodology using qualitative and participatory approaches.^1^ There were 51 participants at the AAR, with representation from NWDoH, WHO, Non-Governmental Organisations Right to Care and Aurum Institute. The AAR began with introductory presentations on the AAR methodology, the objectives, the agenda and an epidemiological overview of COVID-19 in NWP. The AAR focusing on five functional areas was conducted in five working groups. Functional area leads, working with members of their teams, developed presentations that covered the milestones of the response, what went well and what went less well and reasons thereof. The presentations also suggested what should be done to improve the response. This presentation formed the basis for the facilitated discussions in the groups. Groups worked in their pre-identified functions, there were opportunities to share and undertake cross-learning with other functional groups throughout the process. The groups focused on the following five key areas:

1. Coordination, leadership and governance.
2. Epidemiology, surveillance and laboratory.
3. COVID-19 case management and continuity of essential services.
4. Risk communication and community engagement.
5. COVID-19 vaccination.

Each focus area considered the following in detail:

- What was in place before the response: This provided the baseline regarding structures in place to support a health response including the systems, plans, policies, resources, among others.
- What happened during the response: This identified key milestones, achievements and activities in the health response and developed timelines of the event in order to establish a common operating picture among participants and agree on key facts related to the emergency being reviewed.
- What went well, less well and why: The working groups collectively analysed the actions taken to respond to identify the best practices and challenges encountered during the response, their impact on the response and why they occurred.
- What can be done to improve response: Working groups identified and developed key activities to address the best practices and challenges.
- Consolidation: The final session involved the collective prioritisation of activities identified during the AAR workshop. Working groups gave feedback on their key priority activities. Finally, the groups together decided how the activities identified would be taken forward, including the immediate next steps for ensuring implementation.

### Ethical considerations

The AAR that was conducted as part of routine health service quality appraisal that did not require ethical clearance as the review did not use or gather personal or sensitive data or involve experiments with humans or animals. Personal identifiers of the AAR participants are excluded from the report.

## Results

Findings were classified into five function areas presented in Table 1.

**TABLE 1:** Results of the after action review.

Function	Best practices	Challenges
Coordination, leadership and governance	The timely establishment and activation of a provincial intergovernmental coordinating structure and Strategic Management Committee led to effective coordination, decision-making and resource mobilisation. Establishment of provincial and district nerve centres facilitated implementation of the response plans. The development and updating of response plans based on the epidemiological situation led to buy-in from all parties involved and the prepositioning of essential goods. Cross-border collaboration led to the timely detection of imported cases and prevented the spread of cases across borders.	Lack of pre-existing Emergency Operation Centres (EOCs), Incident Management Systems, inadequate pandemic preparedness and operational readiness to respond to emergencies resulted in delayed response. Massive repurposing of departmental personnel and facilities in the absence of a proper pre-existing plan resulted in gaps that hindered continuity of other essential health services. Weak coordination across the different workstreams of the nerve centre resulted in silos and duplication of efforts at times.
Epidemiology, surveillance and laboratory	Daily analysis of surveillance and epidemiological data and broadly disseminating situation reports updated all stakeholders and aided response activities. Improved and more effective internal and external communication led to improved surveillance data quality, a timelier response and increased ownership of surveillance data. The use of the existing ward-based outreach response approach enhanced provincial readiness and community-based surveillance activities. The presence of an integrated laboratory information system enabled real-time dissemination of results through short messages system (SMS) The availability of mobile laboratory testing increased uptake and reach to all areas including those previously inaccessible.	Inadequate integration of surveillance systems resulted in incomplete data linking and harmonisation. Inadequate surveillance capacity at district and sub-district levels affected the quality of surveillance data and the optimal implementation of surveillance activities. During the peak of the pandemic, the laboratory delayed turn-around time delayed the isolation of positive cases.
COVID-19 case management and continuity of essential services	Early dissemination of case management protocols to facilities, coupled with training support on guideline implementation ensured standardisation of treatment regimens and case management across the province. Establishment of the response teams, Infection Prevention and Control (IPC) and emergency medical services at district and subdistrict levels, strengthened local case management capacity. Establishment of field hospitals increased the available bed capacity. Prepositioning and restocking of drugs and medical supplies in the response resulted in the early commencement of treatment.	Staff shortages and inadequate psychosocial support led to work overload, staff fatigue and burnout. Lack of existing strategic supply chain plans resulted in delays in procurement and delivery of supplies. Miscommunication between IPC and Occupational Health and Safety (OHS) resulted in poor quality personnel protective equipment (PPE) and irrational PPE use by healthcare workers. Case referrals were delayed because of unclear referral pathways.
Risk communication and community engagement	The availability of a media and communication plan that involved both conventional and social media platforms guided the scope of intervention. Involvement of community leaders and stakeholders in planning, community mobilisation and response increased awareness at all levels and mobilisation of resources and ownership. The use of multiple communication channels in the dissemination of information helped target different audiences, empower people with real-time information on how to prevent and protect themselves from infection and encourage health-seeking behaviours.	Insufficient communication tools and late deployment affected full implementation of planned activities. Inadequate coverage of the target audience because of the failure to translate IECs at the provincial level into local languages. Ineffective and inefficient system to deal with myths regarding COVID-19 and vaccination resulting in poor uptake of preventive measures.
COVID-19 vaccination	Inter-sectoral collaboration improved coordination and dissemination of information. Early expanded vaccination access through outreach programmes, demand creation, health promotion and mobilisation were achieved through the involvement of religious leaders, traditional leaders, higher learning institutions, political leadership and other departments during the early phase of the mass COVID-19 vaccination. Daily or weekly virtual meetings to track progress improved vaccine accountability. Optimal stock management using existing systems such as Stock Visibility System (SVS) Schedule-6 Register, RX System improved the utilisation and accountability of vaccines and response materials. Monitoring and supervision by the vaccination team led to efficient and quality implementation of the vaccine programme.	Lack of transportation of vaccines and vaccinators to outreach sites resulted in delays. Poor network connectivity and coverage at some vaccination sites and unreliable power supply compromised the delivery of the programme, quality of the vaccines, equipment and service delivery. Rapid evolution of the vaccination programme without sustained training updates could have resulted in practice error, programmatic adverse events and poor adverse events following immunisation (AEFI) data.

*Source:* COVID-19 resources and news portal. https://sacoronavirus.co.za

COVID-19, coronavirus disease 2019; IEC, Information Education and Communication.

The governance, leadership and coordination function identified issues related to consensus building, ownership and leadership of the response by the province, disaster preparedness and response planning and incident management systems. It also identified strategies used to mobilise and organise all resources (governments and partners) for effective outbreak containment.

Disease data flow and harmonisation (completeness and timeliness); proper sample collection, transportation and timeliness of results were discussed in the surveillance and laboratory function, to provide reliable information for proper planning and timely public health actions. The function identified the need for strengthening district laboratory capacity.

Case Management and Continuity of Essential Health Services function highlighted the prompt deployment of experts, training, guidelines and standard operating procedures (SOPs) guided clinical management. Furthermore, the importance of preventing spread through Infection Prevention and Control (IPC) measures was stressed.

Risk Communication and Community Engagement (RCCE) function identified the existence of a media and communication plan by the NWDoH as having played a key role during the outbreak. Effective coordination, media engagement and deployment of Information Education and Communication (IEC) materials were also good practices identified during the outbreak.

The COVID-19 vaccination function identified intersectoral collaboration and outreach programmes as one of its best practices during the outbreak, attributing this to the early submission and approval of budget lines, as well as partners’ and the government’s dedication. Some of the challenges identified included non-compliance to vaccination and transport and connectivity issues.

## Discussion

The COVID-19 pandemic swept across the globe, challenging nations, regions and communities to respond swiftly and effectively to a crisis of unprecedented proportions. As we delved into the AAR of the COVID-19 response in the NWP, we critically examined key dimensions that shaped the course of events and outcomes. This encompassed a comprehensive analysis of the province’s coordination, leadership and governance strategies; the pivotal role of epidemiology, surveillance and laboratory services; the intricate interplay between COVID-19 case management and the maintenance of essential services; the efficacy of RCCE efforts and the pivotal drive towards COVID-19 vaccination.

The COVID-19 pandemic has underscored the critical importance of comprehensive pandemic preparedness and response strategies.^2,3,4^ This current AAR like the Lancet Commission on lessons for the future from the COVID-19 pandemic provides a comprehensive framework to analyse the best practices, challenges, previous actions and the lasting legacy of the pandemic.^2^ The authors sought to distil key insights from the Commission’s findings, shedding light on the global response to the crisis and charting a course for future pandemic readiness. We underscored the importance of swift coordination and innovative governance strategies in responding to a pandemic. The highlighted best practices and prioritised actions provide a roadmap for future pandemic preparedness,^2,3^ while the legacy projects emphasise the significance of collaboration, data management, technological advancement and sustained monitoring for robust public health responses and crisis management. The pandemic reinforced the necessity of collaborative efforts between public health, government, science and communities. Successful responses hinged on multidisciplinary partnerships and information sharing.^5,6^ This thorough review highlighted the value of adaptable governance, unambiguous leadership and efficient cooperation in the event of a pandemic. The suggested actions – both short-term and long-term – highlight the value of ongoing preparation, cross-sectoral cooperation and technical improvements for potential pandemic responses. The legacy initiatives provide a blueprint for strengthening resilience and adaptation in health systems after the COVID-19 era by providing concrete examples of the results of strong cooperation, creative solutions and thorough monitoring.^2,6^ This assessment of epidemiology, surveillance and laboratory functions underscores the significance of data harmonisation, timely results and community-based approaches in effective pandemic response. The recommended actions, spanning immediate to long-term, emphasise strengthening communication platforms, expanding testing capabilities^7^ and enhancing training and funding for sustained improvements. The legacy projects exemplify the lasting impact of real-time information dissemination and crisis management frameworks, providing a foundation for future disease surveillance and outbreak response efforts. Our evaluation of case management and essential service continuity highlighted the importance of readiness, proper training and collaboration in pandemic response.^3^ The recommended actions, ranging from immediate to long-term, underscore the need for integrated care, staff well-being and governance improvements to build resilient health systems. The legacy projects showcase sustainable collaborations, functional response centres and capacity enhancements for future preparedness and response efforts. During the outbreak, the RCCE function was crucial, with a media and communication plan directing interventions via traditional and social media platforms. Participation of community leaders and stakeholders boosted awareness and resource mobilisation and a variety of communication channels equipped people with up-to-the-minute preventive knowledge. Lack of resources that affected execution, poor translation of materials into local languages and a dearth of techniques to deal with myths and misinformation were among the difficulties. Establishing RCCE structures, decentralising procurement, and activating community engagement teams are immediate steps that must be taken while switching to digital platforms. In the long run, COVID-19 legacy projects that aim to strengthen efficient risk communication and community involvement include scalable call centres, assuring committed funding and maintaining nerve centres and call centres. The COVID-19 vaccination function, which highlighted intersectoral cooperation and outreach initiatives as crucial practices, marked a turning point in the pandemic with authorised vaccines. Non-compliance, transportation and connectivity concerns were problems. Inter-sectoral cooperation, early extended vaccine access through outreach programmes including various leaders and institutions, virtual progress tracking, mobilisation at vaccination sites, ideal stock management and careful monitoring were key effective practices. Setting supervision visit goals, converting vaccination safety protocols into clinical governance for quality control, looking into public-private sector collaborations and improving computer literacy are all immediate tasks. Long-term initiatives include the expansion and upkeep of cold-chain equipment, a steady power supply and dependable internet access. Important legacy projects illustrate a diverse approach to successful COVID-19 immunisation, including enhancing clinical governance, resource pooling and active vaccination oversight.

### Recommendations

On the basis of findings from this review, the following recommendations are made:

1. Establish multi-hazard Emergency Operation Centres (EOCs) and incident management systems for coordination of outbreak response in standardised manner integrating responsibilities for health workers into NWDoH incident management plans to address this capability consistently.
2. Develop a robust integrated surveillance system (IDSR) and scale up the use of digital innovations to increase the timeliness of detection and response.
3. Develop strategies and processes to identify risks and protect the health and safety of responders by establishing flexible mechanisms for the rapid mobilisation of trained healthcare workers if surge capacity for case management is required, as well as mechanisms to rapidly establish community treatment centres, including surges for supplies, equipment and personnel, drawing lessons from the devastating effects of the COVID-19 pandemic.
4. Establishment of RCCE structures at all levels that would strengthen linkage between surveillance, case management, risk communication with health promotion.
5. Transition and integration of COVID-19 vaccination programme into the routine Expanded Vaccination programme and guide vaccine safety structures to serve as clinical governance structures (pharmacovigilance) to strengthen quality assurance services.

## Conclusion

The COVID-19 outbreak has exposed the importance of having adaptable health systems. Participants stressed the importance of strong governance and coordination, strong partnerships and healthcare personnel readiness and availability for case management and vaccination. The review highlights the importance of surging epidemiological, laboratory and communication response to address the appropriate public health requirements of significant reserve capacity in peacetime to allow rapid expansion when called upon in an emergency. The recommendations hold lessons for the ongoing COVID-19 pandemic and future responses to emerging infectious diseases. It is critical that the activities identified and best practices should be institutionalised to ensure continuity.
